# Letter from Dr. Leake

**Published:** 1890-05

**Authors:** 


### Correspondence.

### LETTER FROM DR. LEAKE.

DALLAS, TEXAS, April 26, 1890.

*Editor Daniel's Texas Medical Journal:*

SIR:—Recognizing your fitness to decide questions of philology and the important fact that as Chairman of the Publishing Committee of the State Medical Association, such questions may arise in the papers read before the Association and may be settled by your authoritative supervision, I desire to submit the following for your consideration:

1. Should not the term "laparatomy"—(Greek, the "flank" and "section")—be substituted for lapar*o*tomy—the term almost universally employed; since for no apparent reason the English O, corresponding with the Greek omikron, replaces the English A, corresponding with the Greek alpha—the ultimate letter of the first element of the compound term?

2. Is not the term "corpor*e*al endometritis" invariably used by all medical writers, so far as I discover, stilted nonsense? The distinction intended to be made is between an endomitritis of the body of the uterus and that of the cervix, or neck. But is this accomplished by employing the high-sounding term corpor*e*al? The latter is somewhat an abstract term, coined to denote the bodily, as distinct from the spiritual part of man. This should be its specific, and therefore, exclusive, import. It is true, the meaning has been questionably extended, to signify the deeper, as opposed to the external parts of the body—for example, "corporal punishment," not corpor*e*al punishment"—and which, hence, might, on superficial thought, be tortured into an application of meaning to the inner mucous membrane of the uterus. Evidently, however, the specific distinction is meant as above stated; consequently, it appears, that the term should simply be "corporal endometritis," that is, an endometritis of the tangible, anatomical body of the uterus; since there can be no such ludicrous thing as an incorporeal, or spiritual endometritis—the linguistic antithesis; at all events, not "in this vale of tears."

3. Will you please inform me, why, on simply referring papers read before the Association, to the Publishing Committee, as they are often actually done by their titles or headings, they are said to be referred by their "captions?" By what vested authority shall we divert a term from its specifically legal, to a medical signification? Please consider the etymology of the term. It is not from "caput"—"the head"—at all, but from "capio"—"I take," or "I seize,"—and hence, is confined to its legal signification, because it means "that part of a legal instrument which shows where, when, and by what authority it was taken, found, or executed." Its extension of meaning to legal documents of any character whatsoever, is a mild perversion of original meaning, readily tolerated, since the legal relation is still retained. What right, therefore, have medical or other assemblies to its employment, while restricting its application to the specific business of such assemblies; more particularly, as is reserved for them the highly expressive and euphemistic term—"title," or "heading?"

By publishing this communication, together with satisfactory answers, you may confer a benefit on many members of the medical profession in Texas, but one not more to be desired by others than by Yours respectfully,

HENRY K. LEAKE, M. D.

[In transmitting the above communication, the author writes: "I do not want to appear hypocritical, since I make no claims whatever to being a linguistic autocrat, or teacher, but I feel assured that some one will, in the near future perhaps, call the attention of the profession to the same particulars which I have mentioned; not early enough, however, for them to be admitted before the appearance of my paper in the published Transactions, wherein I have anticipated such critics."

This is the *raison d'être* of the communication, presumably.]

The Doctor is correct in all of his objections. "Corporeal endometritis" is, certainly "stilted nonsense;" it means literally, an inflammation of the endometrium, *having a body!* But, "corporal endometritis" is also objectionable, the adjective being superfluous. To say "endometritis," is sufficient; and we understand that it means an inflammation of the inside of the womb; if it is wished to discriminate between inflammation of the womb proper, and inflammation of the neck of the womb, the word "cervicitis" or "endo-cervicitis" (as the case may be) fills the requirement. But the Doctor is wrong when he says that papers are "referred by caption." Some papers are *read* by caption and referred. To say "read by title" would, perhaps, be more "euphemistic;" but, although "caption" is *not* derived from "caput, the head," from time immemorial it has been a custom in journalism to call the "heading" or title of an article the "caption." "Custom, sactioned by good usage, becomes a law." Besides, there are exceptions to all rules; and it is respectfully submitted that there is as much sense and justice in twisting "caption, from capio, to seize," so as to refer to the heading or title of an article, as it is to make the personal pronoun "you" (second person singular) the nominative to a verb, which agrees with a plural nominative, as "you are." "You *is*" would be strictly proper, but "you are," we presume, is more "euphemistic," as well as more euphonious. The two cases are not parallel, but the latter is cited to illustrate the effect of "custom sanctioned by long usage."

With regard to laparotomy—doubtless it should be "laparatomy"—and it would be hard to say why it was ever written otherwise. But—it is another instance of technical error perpetuated by custom and sanctioned by usage. To disturb it now, would be to upset the terminology of a long list of compound words—and we should have to say "phleb-atomy," "ten-atomy," "tracheatomy," etc., etc.

We readily acquit the Doctor of "hypocrisy;" no one who knows him would ever suspect him of being "hypocritical," and we are a little surprised that he should make such an intimation. We know him to be incapable of hypocrisy; but in the light of the above objections, taken in connection with his own well-known high-flown style of diction, may he not be said to be a little—just a little—hypercritical? In fact—judging from his published papers and his private letters to us, we have thought him decidedly euphuistic,—and not unlike Scott's Sir Piercie Shafton, the prototype of that class of ornate writers.

